# Do Individual Females Differ Intrinsically in Their Propensity to Engage in Extra-Pair Copulations?

**DOI:** 10.1371/journal.pone.0000952

**Published:** 2007-09-26

**Authors:** Wolfgang Forstmeier

**Affiliations:** 1 Department of Animal and Plant Sciences, University of Sheffield, Sheffield, United Kingdom; 2 Department of Behavioural Ecology and Evolutionary Genetics, Max Planck Institute for Ornithology, Seewiesen, Germany; University of Exeter, United Kingdom

## Abstract

**Background:**

While many studies have investigated the occurrence of extra-pair paternity in wild populations of birds, we still know surprisingly little about whether individual females differ intrinsically in their principal readiness to copulate, and to what extent this readiness is affected by male attractiveness.

**Methodology/Findings:**

To address this question I used captive zebra finches (*Taeniopygia guttata*) as a model system. I first measured female readiness to copulate when courted by a male for the first time in life. Second, I conducted choice-chamber experiments to assess the mating preferences of individual females prior to pair formation. I then paired females socially with a non-desired mate and once they had formed a stable pair bond, I observed the inclination of these females to engage in extra-pair copulations with various males. Females showing a high readiness to copulate when courted by a male for the first time in life were much more likely to engage in extra-pair copulations later in life than others. Male attractiveness, as measured in choice tests, was a useful predictor of whether females engaged in extra-pair copulations with these males, but, surprisingly, the attractiveness of a female's social partner had no effect on her fidelity. However, it remained unclear what made some males more attractive than others. Contrary to a widespread but rarely tested hypothesis, females did not preferentially copulate with males having a redder beak or singing at a higher rate. Rather it seemed that song rate was a confounding factor in choice-chamber experiments: song attracted the female's attention but did not increase the male's attractiveness as a copulation partner.

**Conclusions/Significance:**

Intrinsic variation in female readiness to copulate as well as variation in the attractiveness of the extra-pair male but not the social partner decided the outcome of extra-pair encounters.

## Introduction

The discovery that extra-pair paternity is both frequent and widespread among socially monogamous birds [1]–[4] had a great impact on our understanding of avian mating systems [5], [6]. Many studies have looked at how the occurrence of extra-pair paternity relates to variation in male characteristics. This has been done by either comparing cuckolded males with non-cuckolded males, or, where paternity could be assigned to extra-pair offspring, by comparing cuckolded males with the males cuckolding them. Why some females engage in extra-pair copulations while others do not, has most frequently been attributed to social circumstances, i.e. to being paired to a favoured vs. an unfavoured male. In contrast, the possibility that individual females may differ intrinsically (independent of social circumstances) in their propensity to seek extra-pair copulations has received only little attention.

Recently, there has been a growing interest in consistent individual differences in behaviour that are maintained across different contexts, which are often referred to as ‘behavioural syndromes’ or ‘personality differences’ [7], [8]. Most empirical studies have looked at behavioural traits other than sexual behaviour (e.g. boldness, see [7]). A few studies that have tested whether fidelity (rate of extra-pair paternity) of individual females is repeatable across different mating situations (i.e. with different social partners), but they did not find significant differences between females [9], [10]. However, despite fairly large sample sizes, field studies might often lack the statistical power required to detect individual differences in female promiscuity. This is primarily because field conditions normally do not allow us to observe the readiness of females to engage in extra-pair copulations, but rather limit us to drawing conclusions about female behaviour from patterns of paternity. Given that patterns of paternity in the wild will be influenced by many other factors such as variation in male attractiveness, availability of extra-pair males, male mate guarding behaviour, or competitiveness of male sperm [1]–[4], demonstrating an effect of female personality has remained elusive.

A first step towards overcoming these difficulties is to observe the behaviour of birds under controlled conditions in captivity [11], [12]. Laboratory settings offer two main advantages. First, the inclination of females to engage in extra-pair copulations can be observed directly [11] and does not have to be inferred from paternity patterns. Second, variation in male attractiveness can be measured in independent choice tests, and can be controlled for statistically or experimentally on an individual basis (i.e. simultaneously controlling for variation in female mating preferences; [13]). Such controls are not feasible in the wild, and so captive studies offer unique potential to investigate questions that are beyond the scope of field studies. Arguably, the unnatural circumstances imposed by captivity and domestication may prevent direct generalization of results to wild populations. While the controversy about generalizability from captivity to the wild may be hard to resolve, captive studies on female promiscuity may also serve an additional function: they encourage taking a closer look at the behavioural signs of female readiness to copulate, which may also be feasible in some field systems.

Zebra finches (*Taeniopygia guttata*) form exceptionally stable, life-long pair bonds both in the wild and in captivity [14]. In captivity, these pair bonds persist even under extended physical separation [15]. Despite such pronounced social monogamy, zebra fiches readily engage in extra-pair copulations. Although different rates of extra-pair paternity have been reported from the wild (2.4% of 82 offspring; [16]) and captivity (28% of 278 aviary-bred offspring; [17]), similar frequencies of extra-pair courtship have been observed in the wild (approx. 0.5 courtships an hour per male; Fig. 1 in [18]) and in captivity (0.46 courtships an hour per male; [19]). These courtships lead to similar frequencies of unsuccessful extra-pair copulation attempts (18% vs. 17% of courtships in the wild and captivity, respectively) and to similar frequencies of successful extra-pair copulations (2.4% vs. 3.3% of courtships; [18], [19]). Hence the frequencies of extra-pair display, copulation attempt, and female rejection or acceptance observed in aviaries seem similar to the natural situation.

**Figure 1:**
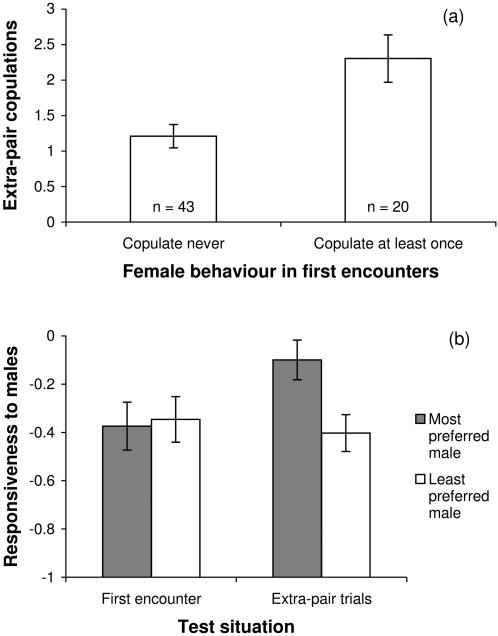
Individual consistency in female copulatory behaviour. (a) Mean number ±SE of extra-pair copulations performed by 63 females in experiment 3, depending on whether females had copulated or not when encountering males for the first time in life (experiment 1). (b) Mean sexual responsiveness ±SE of 63 females shown towards the males they preferred the most and the least, respectively, during choice-chamber tests. Females were more responsive towards preferred males (most vs. least preferred out of four males in a choice chamber), but only during extra-pair mating trials (experiment 3), not when females met them for the first time (experiment 1; first encounters). Responsiveness is measured on a scale from -1 (strong rejection) to +1 (strong inclination to copulate).

Houtman [11] was the first to measure the inclination of individual females to engage in extra-pair copulation under strictly controlled laboratory conditions. She tested 18 female zebra finches for their mating preferences in choice-chamber experiments, and then randomly assigned a partner to these females. When the females initiated a clutch with their partner, they were given the opportunity to engage in extra-pair copulations with (1) a male they had previously preferred as indicated by the amount of time spent near that male, and (2) a male they had not preferred. Six of the 18 females (33%) copulated with an extra-pair male that they had previously preferred whereas none copulated with the male they did not prefer in the earlier choice trials. Houtman [11] also found that a male's song rate was the best predictor of his attractiveness, i.e. his average success in attracting females in choice experiments. So she concluded that song rate was an indicator of male quality, and that females were seeking genetic benefits from copulating with these high-quality males. These conclusions have become widely accepted, as judging from the many citations of her work in the scientific literature. Nevertheless, this much regarded pioneering work by Houtman has never been followed up, thus leaving open three main questions, which shall be addressed in the present study:

1. **Extent of female variation**: Are there intrinsic differences in female readiness to copulate which could explain why some females engage in extra-pair copulations and others do not? Earlier I showed that sexually inexperienced females (when courted by males for the first time in life) differ very consistently in their readiness to copulate [12]. However, it remained unclear whether this variation was context specific (first encounter of a potential partner) or would generalise to other contexts (i.e. inclination to engage in extra-pair copulations). Hence, a main goal of the present study is to see whether a female's sexual fidelity towards her social partner can be predicted from the way she reacted to males when courted for the first time in her life. Consistent variation in general readiness to copulate would affect individual lifetime reproductive strategies and, if heritable, it would be particularly relevant for our understanding of evolutionary changes in mating systems.
2. **The effect of the partner**'**s phenotype**: Does the phenotype of a female's social partner affect her decision to engage in extra-pair copulations? Specifically, are females less likely to engage in extra-pair copulations when paired to a relatively attractive male, i.e. a male that most females spend relatively much time with in choice trials? This question has implications for interpreting those studies which compare cuckolded with non-cuckolded males.
3. **The effect of the extra-pair male**'**s phenotype**: Does a male's attractiveness (measured in the choice chamber) affect his success in obtaining extra-pair copulations, and if so, which component of his phenotype makes a male attractive? It needs to be tested whether females prefer to copulate with males high in song rate, or whether song rate is only a confounding factor when it comes to measuring attractiveness in a choice chamber. The same question arises with regard to male beak colour (orange vs. red), which also has been claimed to reflect male attractiveness and condition in zebra finches [20], [21], but see [13].

Here I address these questions by quantifying intrinsic female variation in readiness to copulate, and the relative importance of the attractiveness of the extra-pair male vs. that of the social partner in the occurrence of extra-pair copulations. In addition, I test whether male song rate, which has been interpreted as reflecting genetic quality [11], [22], affects male attractiveness in extra-pair encounters where females are thought to seek good-gene benefits. To do this I used the same experimental approach as Houtman [11] with an increased number of females, and assessed the behaviour of these females repeatedly under different circumstances. I measured baseline male song rate and female sexual responsiveness (i.e. the general readiness of females to engage in copulation) as individual characteristics (experiment 1), and male attractiveness and female mating preferences in choice tests (experiment 2) before conducting the extra-pair mating trials (experiment 3).

## Materials and Methods

The subjects of this study were 104 male and 104 female zebra finches originating from a large captive population maintained at the University of Sheffield, where all experiments were carried out. The birds were split into 13 experimental groups consisting of eight males and eight females. From the age of 35 days onwards (clearly before the onset of sexual activity) sexes were always kept in separate cages except for the brief experimental encounters described here (experiments 1 and 2). During the third experiment heterosexual pairs were kept in individual cages.

**Experiment (1): First encounters to quantify the baseline sexual responsiveness**: Each female experienced being courted by males for the first time in their life by being exposed to one five-minute encounter with each of the eight males of her experimental group in a randomly chosen order (conducted on eight consecutive days, one test per individual per day). I scored her sexual responsiveness (see below) and measured male song rate for every trial.

**Experiment (2): Choice tests to assess preferences**: The eight males of each group were split into two subsets (A and B) to be used as stimulus males in a choice chamber with four arms. Each female was tested in the choice chamber three times, once with one subset of four males and then twice with the other subset (ABB or BAA), which was done to assess the repeatability of preferences (repeatability of preferences in this experiment was reported in [13]). The second trial was conducted 6±7 days (mean±SD) after the first (minimum 3 days), and the third trial 64±18 days after the second (minimum 24 days). I measured the time in seconds a female spent next to each male. For the purpose of the present study, measurements from repeated trials (BB or AA) were averaged.

**Experiment (3): Extra-pair trials**: Females were paired socially to a male they had ranked second or third out of the four males in a choice test. Each time when initiating a clutch with their partner (i.e. on two occasions) females were given the opportunity to engage in extra-pair copulations with the males they had ranked first and fourth (details below). Social pairing and extra-pair testing was first done with one subset of four males and then repeated with the other subset.

The present study focuses on the behaviour of females in experiment (3), but this is done in relation to how the same females behaved towards the same males in the two preceding experiments. Most importantly, experiments (1) and (2) measured the sexual behaviour of birds before pair formation, while experiment (3) was conducted with socially paired birds. Experiments (1) and (2) have been analysed previously for different questions (see [12], [13]). Hence, in the following, I only give a brief summary of the design of these two experiments. More details on experimental conditions as well as rearing and housing conditions can be found there.

### Female responsiveness, male attractiveness, song rate, and beak colour

The sexual responsiveness of females was judged during first encounters with males (experiment 1) based on the occurrence of positive (tail quivering, beak wiping, approaching, ritualised hopping) and negative (aggression, threat display, beak fencing, fleeing) cues [12]. Based on the frequency and intensity of these behavioural cues, subjective scores of female responsiveness were given on a five-grade scale reaching from a clear rejection of the male (−1) to a clear intent to copulate (+1). Intermediate scores (−0.5; 0; +0.5) were given if a mixture of both positive and negative cues occurred or if either positive or negative cues were only weakly expressed. Because successful copulations decrease the female's readiness to copulate again within the short time of the trial, any cues occurring after successful copulation were disregarded. A change in the opposite direction (i.e. an initial rejection turning into acceptance of the male) was rarely observed and only ever happened within the first minute of male display. So except for the very beginning of a trial, rejection behaviours remain stable for at least one hour (personal observations), and therefore seem a valid expression of dislike rather than part of a mating ritual. Trials without any cues of female responsiveness were treated as missing values. The occurrence of successful copulations (cloacal contact) was scored as yes or no for all trials.

Using a stopwatch I measured male song rate as the number of seconds of song directed towards the female during the five minutes of the trial. Undirected song [14] occurred very rarely and was not measured. Values were square-root transformed and averaged for each male over the eight females encountered (repeatability *R* = 0.59; *F*_103,728 = _12.7; *P*<0.0001). Male and female beak colour was scored subjectively on a scale ranging from 0 (light orange) to 6 (dark red) using Munsell colour chips. This was done on three occasions over a period of six months: approximately three months before the start of experiments, during pair-wise encounters (experiment 1), and, another three months later, during choice-chamber tests (experiment 2). The three scores were averaged for each individual (males: repeatability *R* = 0.49; *F*_101,204 = _3.9; *P*<0.0001; females: repeatability *R* = 0.64; *F*_99,200 = _6.3; *P*<0.0001; data on some individuals were incomplete). On the same three occasions I measured female body mass (repeatability *R* = 0.81; *F*_99,200 = _13.7; *P*<0.0001).

In experiment (2) male attractiveness was measured in choice-chamber trials as the proportion of time a female spent next to a particular male of the total time spent next to any of the four males (expected value = 0.25; [13]). Attractiveness scores were averaged across the eight females judging a male (giving each female equal weight). For some analyses this measurement was split into two components: attractiveness to a focal female vs. average attractiveness to the seven other females.

### Pair formation and extra-pair trials

The design of experiment (3) is best illustrated by an example (Table 1). Assume a female had first been tested in the choice chamber with the male subset A and later with subset B and had ranked these males in terms of how much time she spent next to them in the order A_1_>A_2_>A_3_>A_4_ and B_1_>B_2_>B_3_>B_4_ (results of experiment 2). Approximately one month after the last choice test, the female was paired socially to either male B_2_ or B_3_. This assignment of partners is meant to reflect a situation in the wild, where females also will often end up paired to a male other than their most preferred (because the number of highly attractive males is limited). As will be shown below, stable pair bonds are formed even under such a no-choice situation (see: “Stability of social pair bonds”). Pairs were housed in individual cages and were provided with a nest box. On the day the first egg was laid (i.e. when most extra-pair copulations happen in the wild; [18]) the partner was removed (out of sight) and replaced by the extra-pair males B_1_ and B_4_ (five minutes each in quick succession, order randomised). This procedure was repeated when the pair was allowed a second breeding attempt (on average 41±14 days later; no pair was allowed to raise any offspring), but this time the order of presenting the extra-pair males was reversed (to balance order effects). All pairs were then split up, keeping the former partners in separate rooms. After at least 80 days of separation, females were again paired, this time with either male A_2_ or A_3_. As before, females were allowed to make two breeding attempts (28±8 days apart) and four extra-pair trials were conducted (e.g. first clutch: A_4_ followed by A_1_ and second clutch: A_1_ followed by A_4_). Hence, in total, every female had eight extra-pair trials spread over four breeding attempts involving two different partners and four different extra-pair males. Males participated in 7.1±2.8 extra-pair trials depending on how many females had ranked them highest or lowest.

**Table 1:** Assignment of social partners and extra-pair males to females for copulation trials.

Trial #	Pairing	Social partner	Clutch	Extra-pair male
1	1	B_2 or 3_	1	B_1_
2	1	B_2 or 3_	1	B_4_
3	1	B_2 or 3_	2	B_4_
4	1	B_2 or 3_	2	B_1_
5	2	A_2 or 3_	1	A_4_
6	2	A_2 or 3_	1	A_1_
7	2	A_2 or 3_	2	A_1_
8	2	A_2 or 3_	2	A_4_

Assignment is based on choice-chamber tests where the female ranked males in the order A_1_>A_2_>A_3_>A_4_ and B_1_>B_2_>B_3_>B_4_ (descending order of time allocation). Clutch denotes the number of the breeding attempt with the given social partner.

Not all females completed the eight tests: Initially 86 pairs were formed, but in the second round only 71 pairs could be formed due to limitation of space. Also, in a few cases it was not possible to follow the above rules of assignment of males (because some males were never ranked second or third, or because of mortality). In these cases, pairs were formed to ensure that all males used as extra-pair males had a partner. The females paired to these males were also tested for extra-pair copulations, but they had to be omitted from some of the analyses if they did not fit the required experimental design. Finally, some females failed to lay eggs, so they were tested towards the end of the experiment (affecting 60 out of 614 extra-pair trials). Female status (laying vs. non-laying) was unrelated to the occurrence of extra-pair copulations (Fisher's exact test: Chi^2^ (1) = 0.3, *P* = 0.72), and so data from these females was pooled with those from females that laid eggs. I found no significant effects of daytime, season, female age or time since pair formation on female responsiveness or extra-pair copulations (not shown).

All trials were video-taped and analyzed for whether successful unforced copulations occurred. Forced copulation attempts by males occurred frequently (in 43.8% of all trials), but they never resulted in cloacal contact, because females resistance was always effective. Hence females clearly controlled the outcome of all unsolicited copulation attempts [12]. Again, I measured female responsiveness and male song rate as described above.

Birds were generally maintained on a standard diet [12], but most pairs received supplementary egg food during some phases of the experiment. However, food supplementation did not seem to affect female responsiveness (unpubl. data).

### Stability of pair bonds

To check whether experimentally enforced pair formation in fact leads to the establishment of a permanent social pair bond [23], I released six groups each consisting of four pairs (four males belonging to the same subset during choice-chamber tests and their assigned partners) into six aviaries. Hence, in this setup, all four females in each group had the opportunity to try to switch to a male they had preferred over their assigned partner. The experiment was done after the first round of pair formation, and after the partners had been together in a single cage for three months (involving two unsuccessful breeding attempts and four extra-pair trials). Aviaries were fitted with a surplus of nest boxes and nesting material. Nest boxes were checked for eggs every day, and observations were also carried out daily to determine which birds ended up nesting together. All six trials were terminated after 12 days.

## Results

In 522 out of 614 (85.0%) extra-pair trials (experiment 3) males displayed towards females and in 295 (48.0%) males attempted to copulate with the female. However, copulation attempts were never successful when the female resisted them (see also [12]), hence female responsiveness scores were strongly correlated with the occurrence of successful copulations (explaining 58% of the deviance in a logistic regression; *N* = 533 trials with responsiveness scores, *Z* = 11.2, *P*<0.0001). Successful copulations occurred in 110 out of 614 (17.9%) trials, and in 57 trials (9.3%) copulations had been preceded by female solicitation (tail quivering). In six trials (1.0%) females solicited, but no successful copulation followed.

### Consistency of female behaviour

There were 63 experimental females that completed both the eight trials of first encounter (experiment 1) and the eight extra-pair copulation trials (experiment 3). Twenty of these females (32%) had copulated with at least one out of the eight males in experiment 1 (i.e. before pair formation). Once socially paired in experiment 3 (on average 10 months later), these 20 females engaged in extra-pair copulations approximately twice as often as the remaining 43 females that had not copulated before pair formation (GLM with binomial errors: Chi^2^ (1) = 12.4, *P* = 0.0004; Fig. 1a). This difference in the rate of extra-pair copulation was because the two types of females differed in their sexual responsiveness scores during extra-pair trials (*t*_61_ = −3.1, *P* = 0.0026) and not because they received different amounts of male song (*t*_61_ = −0.5, *P* = 0.63).

The consistency (between experiments 1 and 3) of individual females in their readiness to copulate (i.e. the apparent generality across contexts shown in Fig. 1a) could have resulted from the fact that the same individual males were used in both experiments. This could happen if the experimental groups of eight males (though they were composed randomly) differed in their mean attractiveness, but also if certain males were particularly attractive within a group. To examine this possibility I focus on the consistency (between experiments 1 and 3) of female responsiveness towards particular males, i.e. attractive vs. non-attractive males within experimental groups. During the extra-pair copulation trials (experiment 3), females were significantly more responsive towards the males they had spent most time with in choice tests (experiment 2) than towards the males they had spent least time with in the choice chamber (paired *t*_62_ = 3.4, *P* = 0.001; right half of Fig. 1b). However, the same females had not discriminated between these particular males some 10 months earlier in experiment 1 (before choice experiments and before pair formation; paired *t*_62_ = −0.4, *P* = 0.68; left half of Fig. 1b) and the difference between the two test situations (i.e. the interaction depicted in Fig. 1b) was significant at *P* = 0.01 (repeated-measures linear mixed-effect model with female identity accounting for 35.3% of the variance in responsiveness). Finally, to exclude variation in male attractiveness between experimental groups as a possible confounding factor I examined the average responsiveness of individual females during extra-pair trials as a function of group identity and of responsiveness in experiment 1. Group identity had no significant effect on extra-pair responsiveness (*F*_10,68_ = 1.35; *P* = 0.22; only 11 out of 13 groups entered experiment 3), while pre-pairing responsiveness still had a significant effect after controlling for group identity (*F*_1,68_ = 8.26; *P* = 0.005). All this taken together shows that the consistent sexual responsiveness of individual females between experiments 1 and 3 (Fig. 1a) was not a consequence of the same individual males being used in both tests, since individual males were not reacted to in a consistent way. Hence, the occurrence of extra-pair copulations could partly be predicted from the female baseline propensity to copulate irrespective of male attractiveness.

Although there was some degree of female consistency in overall responsiveness between experiments 1 and 3, consistency within experiment 3 was not significant. Most importantly, of the 40 females that were unfaithful to their first social partner at least once, only 16 (40%) were also unfaithful to their second social partner, and of the 27 females that were never unfaithful to their first partner, nine (33%) were unfaithful to their second partner (Fisher's exact test: Chi^2^ (1) = 0.3, *P* = 0.62).

These analyses suggest that, in extra-pair trials, female responsiveness was partly consistent within individual females and partly influenced by male attractiveness. To quantify the relative importance of the two variables (i.e. female personality and male attractiveness), I analysed variation in female responsiveness as a function of both male and female identity as random effects (*N* = 533 extra-pair trials with responsiveness estimates): the identity of the female explained 32.7%, and the identity of the extra-pair males explained 19.6% of the variation in female responsiveness (female ID: *F*_81,365_ = 3.3, *P*<0.0001; male ID: *F*_80,365_ = 2.0, *P*<0.0001).

Female responsiveness in first encounters (experiment 1) was unrelated to female body mass (*r* = −0.16, *N* = 104, *P* = 0.11) and beak colour (*r* = 0.09, *N* = 104, *P* = 0.38). Responsiveness during extra-pair trials (experiment 3) was again unrelated to body mass (*r* = −0.12, *N* = 86, *P* = 0.27), but females with redder beaks were more responsive towards extra-pair males, though the effect was not significant (*r* = 0.21, *N* = 86, *P* = 0.053).

### The effects of the partner's vs. the extra-pair male's phenotype

**Figure 2:**
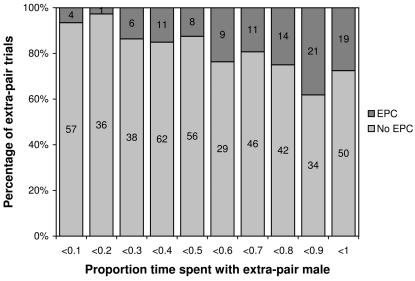
Outcome of 554 extra-pair mating trials in relation to female preferences. The x-axis shows the relative times that females had spent with the extra-pair male vs. their partner during choice-chamber tests conducted before pair formation and is calculated as x = time spent with extra-pair male/(time spent with extra-pair male+time spent with partner). The numbers of trials with and without extra-pair copulations (EPC) are indicated.

There were 554 extra-pair trials (experiment 3) for which the full information was available on how much time the respective females had spent with the extra-pair male vs. their partner during previous choice experiments (experiment 2). Extra-pair copulations were more likely to happen when females had spent relatively more time with this extra-pair male (Fig. 2). These data were analysed in a generalised mixed effects model (lmer, using R 2.4 Free Software Foundation, Inc., Boston, Massachusetts, USA) with binomial error structure using female identity as a random effect and five fixed effects (entered simultaneously): the probability of copulation depended strongly on the time previously spent with the individual extra-pair male (positive effect; *t* = 3.5, *P* = 0.0004), to a lesser extent on the attractiveness of the extra-pair male as judged by the seven other females in choice tests (positive effect; *t* = 3.1, *P* = 0.002), on the order of presentation of the two extra-pair males (first males preferred; *t* = −3.3, *P* = 0.001), but not on the time females had spent with their social partners during choice tests (*t* = −0.05, *P* = 0.96), and not on the attractiveness of their partner as judged by other females (*t* = −0.7, *P* = 0.46). These explanatory variables were not fully independent of each other, creating a potential problem with multicolinearity. However, the time spent with the extra-pair male was only weakly negatively correlated with the time spent with the partner (*r* = −0.21, *P*<0.0001), hence there was sufficient residual variation (1−r^2^ = 0.96) that could have helped explaining female behaviour. Male attractiveness as judged by the focal female was positively correlated with the judgement by the seven other females (*r* = 0.12, *P* = 0.006 for the partners and *r* = 0.32, *P*<0.0001 for the extra-pair males) reflecting some degree of female agreement in preferences. The relatively weak correlation coefficients suggest that multicolinearity was not a major problem.

I extended the above mixed-effect model by including as explanatory variables specific male characteristics that are typically thought to affect male attractiveness, namely song rate (as measured in experiment 1) and beak colour. To maximise the statistical power, I used the pair-wise differences in these characteristics between the social partner and the extra-pair male. However, neither differences in song rate (*t* = −1.0, *P* = 0.34) nor in beak colour (*t* = 0.9, *P* = 0.40) explained the occurrence of extra-pair copulations.

### Song rate and male attractiveness

Male attractiveness during choice experiments (experiment 2; judged by eight females and averaged) was positively correlated with both song rate before pair formation (experiment 1; male averages; *r* = 0.34, *N* = 85, *P* = 0.0015; [13]) and song rate during extra-pair trials (experiment 3; *r* = 0.31, *N* = 86, *P* = 0.004). The two measurements of song rate taken 10 months apart were strongly correlated with each other (experiments 1 and 3; repeatability *R*±SE = 0.60±0.07, *F*_84,85_ = 4.0, *P*<0.0001).

Male copulatory success in extra-pair trials (the proportion of females with which males copulated successfully) was positively related to male attractiveness measured in the choice chamber (experiment 2; GLM with binomial errors, Chi^2^ (1) = 16.8, *P* = 0.00004). Male copulatory success was also positively related to male song rate during extra-pair trials (GLM with binomial errors, Chi^2^ (1) = 11.2, *P* = 0.001). The latter correlation seems trivial, because only males who sing can obtain copulations; those who show no interest in females obtain no copulations even if the female is responsive. Hence, only an analysis of female responsiveness (rather than male copulatory success) can reveal the preferences of females.

**Figure 3:**
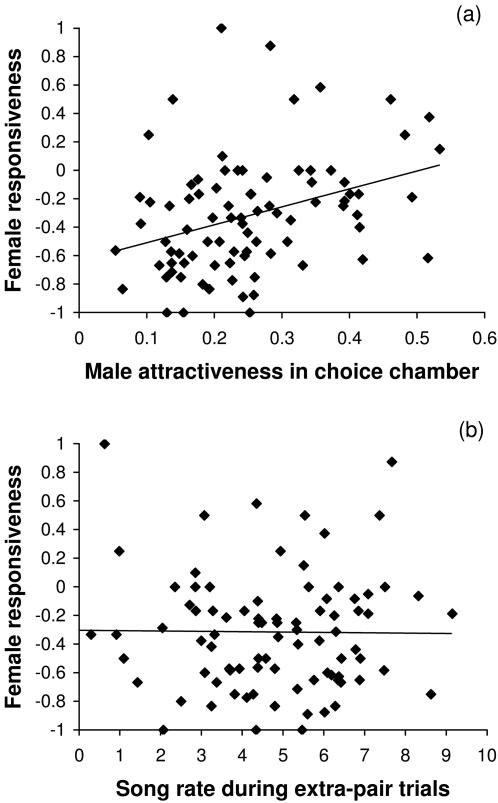
Average female responsiveness during extra-pair mating trials towards 86 males. Explanatory variables are (a) average male attractiveness (averaged across eight females) in the choice chamber, and (b) average male song rate during extra-pair trials. Each data point represents a male. Female responsiveness is measured on a scale from −1 (strong rejection) to +1 (strong inclination to copulate). Attractiveness is measured as the proportion of active time that females spent next to one of the four males in a choice chamber (expected value = 0.25). Song rate is the square-root transformed number of seconds of directed song that males produced during 5-min trials. Lines are fitted regression lines, irrespective of significance.

Female responsiveness towards extra-pair males (male averages across 3.3±1.3 SD females) was again, just like copulatory success, positively correlated with male attractiveness in the choice chamber (*r* = 0.34, *N* = 85, *P* = 0.001; Fig. 3a). However, responsiveness in extra-pair trials was neither related to the song rate of these males during extra-pair trials (experiment 3; male averages; *r* = −0.01, *N* = 85, *P* = 0.91; Fig. 3b) nor to the song rate these males had shown before pair formation (experiment 1; *r* = −0.05, *N* = 84, *P* = 0.63). Female responsiveness in experiment 3 was also not positively related to the redness of a male's beak (*r* = −0.14, *N* = 85, *P* = 0.20; the negative sign stands for a trend towards females preferring orange).

### Stability of social pair bonds

To test whether experimentally enforced pair formation led to stable pair bonds I released six groups of four pairs into six aviaries for a period of 12 days. All 24 pairs remained together (judging from allopreening and sitting in bodily contact; [14]), and 11 pairs were already incubating a clutch of at least four eggs on day 12 of the experiment. One successful extra-pair copulation and one unsuccessful female extra-pair solicitation were seen in a total of nine hours of observation.

## Discussion

### Female individuality

The present study shows that females vary intrinsically in their propensity to engage in extra-pair copulations. However, the study also shows that the attractiveness of the extra-pair male has a large effect on female behaviour, which makes the female-intrinsic effect harder to detect.

In captivity, sexually inexperienced zebra finch females showed highly consistent individual differences in sexual responsiveness towards males, and little if any discrimination between males when courted for the first time in life (here mentioned as experiment 1; results reported in [12]). In that pre-pairing situation, female identity accounted for 65% of the variation in female sexual responsiveness during encounters with a range of males, while male identity accounted for only 7% of the variation in female responsiveness [12]. In the present study, these females were socially paired, and they encountered the same individual males as before (experiment 3). In this situation of females being socially paired, the intrinsic differences in female responsiveness were less conspicuous (accounting for 33% of the variation), and females were more discriminating between males (accounting for 20% of the variation). Nevertheless, it was evident that some of the initial variation in female readiness to copulate was retained between situations. This generality across contexts is consistent with the definition of personality or behavioural syndromes [7]. Individuals that were more responsive when unpaired also had more extra-pair copulations later on than females that initially were less responsive. Hence, I suggest that individual differences in sexual responsiveness shown early in life partly reflect intrinsic variation in female promiscuity. The individual differences in female readiness to copulate partly seem to be maintained across different contexts, i.e. unpaired vs. paired mating status. However, intrinsic differences in promiscuity were not sufficiently pronounced to yield significant repeatability of female extra-pair copulation behaviour across two partners. A likely reason is that only two extra-pair males were offered in each situation, which often may not have been sufficient to stimulate females to copulate. Given the low repeatability of female responsiveness in extra-pair trials and the large effect of male attractiveness (see above), plus the fact that males did not court females at all in 15% of the extra-pair trials, clearly more than four extra-pair encounters per social pairing would have been required to obtain a repeatable copulation record.

The observed rates of copulation attempts by males (57% of trials with display) were higher than found by Burley et al. [19] under aviary conditions (17% of extra-pair courtships), and so were the rates of successful copulation (21% vs. 3.3% of displays). This is likely due to the limited opportunities for extra-pair matings under cage conditions (two 5min-tests per breeding attempt) as compared to frequent encounters under aviary conditions. The frequency of successful copulation (17.9% of all trials) was similar to the 16.7% found by Houtman [11] under very similar cage conditions.

It is possible that female zebra finches, under the unnatural testing conditions in small cages, might have copulated only because they could not escape the males. This concern seems unjustified since: (1) It was usually possible to judge from signs of female responsiveness within the first half minute of a trial whether females would engage in copulation or not. The median latency to copulation was 32sec (*N* = 110), which includes the time males needed to recover from handling. (2) Male aggression against the female never made females more inclined to copulate [12]. (3) Females preferentially copulated with males they had preferred in a choice test, which means that they often resisted the undesired male persistently and then immediately accepted the desired male. Figures 1b and 2 show that females made “sensible” choices during these short extra-pair encounters that were in line with their individual preferences.

Generally, it seems likely that the conditions of captivity and the process of domestication may have had an impact on extra-pair copulation behaviour in this study, and that this might explain the discrepancy in rates of extra-pair paternity observed in captivity and in the wild [16], [17]. However, extra-pair paternity does occur in the wild, and I argue that the observed phenomenon of individuality (here regarding female promiscuity) is unlikely to be solely an artefact of domestication since such individuality (in general) is so ubiquitous in the animal kingdom [7], [24].

It seems unfortunate that there has been so little interest in measuring directly the intrinsic female inclination to copulate. I suggest, that even in some wild settings it may be possible and very rewarding to specifically observe signs of female responsiveness (or the rate of inviting copulations) when courted by (1) the social partner and (2) extra-pair males. A positive correlation between these two measures of responsiveness would argue against mating behaviour being entirely phenotypically flexible (i.e. being fully context-dependent). Given the evidence presented here it seems possible that females that respond more positively to courtship by their own partner would have higher rates of extra-pair paternity in their broods than others simply because they might be less resistant to extra-pair courtship. Note that this pattern would be the opposite of what is commonly assumed, namely that female extra-pair matings would be less frequent when reacting positively to their partner (reflecting “satisfaction” with the genetic quality of the social partner).

### The effect of male attractiveness

In agreement with Houtman [11] I found that females preferred attractive males for extra-pair copulations (Fig. 2). This shows that measurements of male attractiveness obtained with a choice chamber are meaningful in the sense that they translate into a female preference to copulate with these males (see also [25]). Hence, there is natural variation in male attractiveness and this variation can be measured either in a choice chamber (experiment 2) or in extra-pair trials (experiment 3). In contrast, pair-wise encounters of sexually inexperienced birds (experiment 1) seem unsuitable for the measurement of female preferences and male attractiveness [12]. Choice chamber experiments seem to reflect female mating preferences, but given the low individual consistency of female choice and the low between-female agreement [13] much replication is needed to obtain reliable estimates of male attractiveness. Interestingly, the occurrence of extra-pair copulations depended on both the individual preferences of a female and the mean attractiveness of the extra-pair male towards the seven other females of the group. It is likely that the deviation of the preference of a single female from the average preference of the group will partly reflect a measurement error that is initially made by the individual female when assessing male attractiveness during relatively short (3h) choice-chamber trials. Possibly females compensate for this initial measurement error by preferentially copulating with males that were scored most attractive by the other seven females. Such measurement error might also partly explain why the effect of individual preferences on the occurrence of extra-pair copulations was less clear-cut (see Fig. 2) than one might have expected from the data presented by Houtman [11]. However, this could also be because preferences for a social partner and preferences for an extra-pair copulation partner need not coincide. In the choice chamber, unpaired females might select a good partner for raising offspring, while during extra-pair trials they might seek other types of benefits such as good genes.

When females were sexually inexperienced, they did not seem to discriminate between males during five-minute encounters ([12], Forstmeier unpublished data), yet when socially paired, they clearly preferred to have extra-pair copualtions with males which they previously had preferred in choice-chamber experiments (Fig. 1b). This change in female discrimination may have occurred because females required some time to assess male quality (here: 3–6h in the choice chamber) or because females required sexual experience with males (here: the social partner) to learn how to behave in a discriminating way. Alternatively, whether preferences are expressed during short pair-wise encounters may depend on the mating status of the female (unpaired vs. paired). Copulations may have a different function in unpaired females that are searching for a partner and in paired females that might be seeking some kind of indirect benefit.

Rather surprisingly, the present study showed that a female's inclination to engage in extra-pair copulations was independent of her previous judgement of her social partner and also independent of the general attractiveness of her social partner to other females. This might indicate that female promiscuity depended more on the quality of stimulus produced by the courting extra-pair male than the quality of the social partner. This is particularly controversial, since it is widely believed that the attractiveness of the social partner plays a key-role in extra-pair paternity [3]. However, these findings suggest a need for more experimental research on extra-pair paternity using controlled laboratory conditions. Some caution in the interpretation of this result is advised because the experiment was not primarily designed to test for an effect of the attractiveness of the partner, but rather for an effect of extra-pair male attractiveness. The time females had spent (in the choice chamber) with the male that later became their partner (mean±s.d. = 0.17±0.13, i.e. 17%±13% of the total time spent with males) was less variable than the time spent with the males that later functioned as extra-pair males (mean±s.d. = 0.32±0.29), because the latter males had been selected for being the most and the least preferred. However, mean attractiveness of the partner to the other seven females (mean±s.d. = 0.26±0.12) was not less variable than the mean attractiveness of the extra-pair males to the other females (mean±s.d. = 0.26±0.12). So the argument that social partners varied less in attractiveness than extra-pair males cannot explain why the attractiveness of extra-pair males to other females affected the occurrence of extra-pair copulations, while the mean attractiveness of the social partner did not. This finding clearly argues for the extra-pair male's phenotype having a greater effect on the occurrence of extra-pair copulations than the social partner's phenotype.

The finding that a female's promiscuity is independent of the attractiveness of her social partner is inconsistent with results obtained by Burley et al. [17], [19], who found an effect of the partner's ring colour (red vs. green) on the occurrence of extra-pair paternity in aviaries and ring colour had been found to affect male attractiveness. Many factors might be responsible for this difference in results, including the effect of band colours vs. natural variation in attractiveness (no coloured bands were used in the present study), the presence of partner choice in aviaries, the effect of mate guarding by partners, and the effect of post-copulatory sexual selection.

### Song rate confounds measurements of attractiveness

Consistent with earlier findings (Figure 3 in [12]) I found that song rate was positively correlated with copulatory success, but again this was not because females preferred to copulate with males who sang most (Fig. 3b of the present study), but rather because males who never sang to any of the females could not obtain any copulations. Instead, females preferred to copulate with males that were attractive in the choice chamber (Fig. 3a). The differential effect of attractiveness and song rate (Fig. 3a vs. 3b) is surprising, since the two traits are clearly positively correlated. Hence, I suggest that male song attracts the attention of the female in the choice chamber, which leads to a positive correlation between song rate and the time females spend next to males [13]. However, it seems that variation in song rate only confounds measurements of sexual attractiveness made in the choice chamber, since males with a high song rate were no more attractive as extra-pair copulation partners than males with a low song rate. Since Houtman [11] did not show a direct correlation between song rate and female responsiveness either (though it was inferred), it seems that the interpretation of song rate as a good-genes indicator might need a critical reassessment (see [26]). Yet, it still remains to be examined whether song rate might be an indicator of male parental qualities, which could render males who sing the most more attractive as a social partner.

### Conclusions

In their recent review of extra-pair paternity in birds Westneat and Stewart [4] highlight the importance of considering the phenotypes of both members of a pair when trying to understand the occurrence of extra-pair paternity. The present study is the first to specifically investigate the possibility that individual females may differ intrinsically in their propensity to engage in extra-pair copulations. Within the individual female, this propensity seems to vary with the attractiveness of available extra-pair males, but surprisingly not with the attractiveness of her social partner. This suggests that it may be worthwhile examining the possibility that, in the field, male partners may be exerting their influence on paternity via mate guarding, frequent copulation or postcopulatory mechanisms rather than via advertising their genetic quality towards their female partners. Undoubtedly, mating interactions seem to be complex. The best way of dealing with this complexity is to decompose it into its components. For future research I suggest researchers attempt to measure separately (1) intrinsic female readiness to copulate, (2) attractiveness of males, and (3) mate guarding intensity by the pair male. Specifically, I suggest observing signs of female responsiveness to both the partner and extra-pair males. If female responsiveness mostly depends on the relative attractiveness of males, we might expect a negative correlation between a female's responsiveness to her partner and her responsiveness to extra-pair males. If, in contrast, females vary intrinsically in their readiness to copulate, a positive correlation might emerge.
